# Contraception in adolescence: the influence of parity and marital status on contraceptive use in 73 low-and middle-income countries

**DOI:** 10.1186/s12978-019-0686-9

**Published:** 2019-02-21

**Authors:** Carolina de Vargas Nunes Coll, Fernanda Ewerling, Franciele Hellwig, Aluísio Jardim Dornellas de Barros

**Affiliations:** International Center for Equity in Health, Federal University of Pelotas, Rua Marechal Deodoro, 1160, 3rd floor, Pelotas, RS Brazil

**Keywords:** Adolescents, Family planning, Contraceptive, Low-and middle-income countries, Marriage, Pregnancy

## Abstract

**Background:**

There is still a large gap in relation to effectively meet the contraceptive needs and family planning goals of adolescents. Our aim was to describe how having a partner and children impact on contraceptive behavior of sexually active female adolescents from low and middle-income countries (LMICs).

**Methods:**

Analyses were based on the most recent Demographic and Health Surveys and Multiple Indicator Surveys carried out since 2005 in 73 LMICs with available data for sexually active women aged 15–19 years. Modern contraceptive prevalence and demand for family planning satisfied with modern methods of contraception (mDFPS) were estimated among three subgroups of adolescents considering their parity and marital status- not married, married without children, and married with children – at national and regional levels.

**Results:**

Female adolescents who were married with no children presented the lowest median modern contraceptive prevalence in all world regions, ranging from 2.9% in West & Central Africa to 29.0% in Latin America & Caribbean. Regarding mDFPS, the lowest coverage for married adolescents without children was found in West & Central Africa (12.6%), whereas Latin America & Caribbean presented the highest (50.4%). In East Asia & Pacific, not married adolescents were the group with the lowest mDFPS (17.1%). In 12 countries, mDFPS was below 10% among married adolescents without children: Angola, Chad, Congo, Congo DR, Guinea, Mozambique, Niger, Nigeria, and Senegal in Africa, Philippines and Timor-Leste in Asia and Guyana in Latin America & Caribbean.

**Conclusions:**

In most countries, modern contraceptive prevalence and mDFPS were particularly low among married female adolescents without children, which should be considered a priority group for intervention. The findings suggest that social norms regarding marriage and fertility expectations and other cultural barriers have a role at least as relevant as contraceptive availability. All these aspects need to be considered in the design of family planning strategies to effectively increase modern contraceptive use among adolescents everywhere, particularly in conservative contexts.

**Electronic supplementary material:**

The online version of this article (10.1186/s12978-019-0686-9) contains supplementary material, which is available to authorized users.

## Plain English summary

Satisfying the need for contraception is key for reducing child and maternal morbimortality and related social costs of adolescent pregnancies. Although important progress has been made in increasing family planning coverage with modern methods worldwide, there is still a large gap in relation to effectively meet the contraceptive needs and family planning goals of adolescents. We provide a global picture of contraceptive prevalence and the demand for family planning satisfied (DFPS) among sexually active female adolescents from low- and middle-income countries (LMICs), considering their marital status and number of living children. Using data from 73 LMICs, we compared the needs for contraception among three distinct groups of adolescent aged 15–19 years: currently married or in a union - with or without children, and sexually active outside marriage) at the national and regional levels. In most countries, modern contraceptive prevalence and mDFPS were particularly low among married adolescents without children in comparison to the other two groups, which should be considered a priority group for intervention. This suggests that social norms regarding marriage and fertility expectations and other cultural barriers have a role at least as relevant as contraceptive availability. Family planning strategies should consider these contextual factors in order to effectively reach adolescents.

## Background

Universal access to sexual and reproductive health services and rights by 2030, including family planning, is a priority in the global Agenda for Sustainable Development, as laid out in Goals 3 (to ensure good health and promote wellbeing at all ages) and 5 (achieve gender equality and empower all women and girls) [1]. Among the key indicators to track progress towards Sustainable Development Goal 3 are the proportion of women in reproductive age who have their need for family planning satisfied with modern methods, and the adolescent birth rate [2]. Pregnancy and childbirth are the leading causes of death among girls aged 15–19 in low and middle-income countries (LMICs) [3]. In this context, family planning is also crucial for reducing pregnancy-related morbidity and mortality, improving the health outcomes of young mothers and their children, and reducing the related social and economic costs of an early pregnancy [1].

Although important progress has been made in improving coverage of family planning services worldwide [4, 5], there is still a large gap in relation to effectively meet the contraceptive needs and family planning goals of adolescents [1, 5–7]. About 16 million girls aged 15–19 years give birth annually, and for many of them it is an unplanned pregnancy [8]. For many others, an unplanned pregnancy ends in an unsafe abortion [9]. Millions of sexually active girls worldwide (both in a union or not) want to postpone childbearing until finishing school, being employed, or just to space pregnancies [10]. But they lack the knowledge, agency or resources to make decisions regarding planning and timing pregnancy [10].

In some places, restrictive laws and policies continue to affect the ability of adolescents to obtain and use contraception [10]. In many others, even though there are no legal restrictions in providing family planning services to adolescents, the attitudes and opinions of health workers and community leaders have been often found to limit contraceptive information and inhibit contraceptive use before marriage [10, 11]. Besides, even in places where adolescents can obtain contraceptives, social and cultural norms play a major role in repressing their use, making it more difficult for them to achieve the desired family size [10, 12]. Misconceptions about the immediate and long-term side effects of contraceptive methods on their health and future ability to bear children are also commonly reported barriers among female adolescents [10].

Major differences in contraceptive use and family planning goals have been found depending on the women’s marital status and parity. Adolescents who are not married can face several barriers to access and use contraceptives because sexual activity is only considered acceptable within marriage in many settings. Married adolescents, on the other hand, are often under pressure to have a child soon after marriage and end up pregnant at early ages [13, 14]. One third of girls in LMICs are married or in a union before the age of 18, with great variation across countries [14]. Low decision-making power and rigid social norms often results in low use of effective methods of contraception among married female adolescents, although many of them wish to delay first births or space pregnancies [15].

Our aim was to provide a global picture of contraceptive use prevalence and the demand for family planning satisfied (DFPS) coverage among sexually active female adolescents from LMICs, considering their marital status and number of living children. Comparing the needs for contraception among distinct groups of adolescent girls (currently married or in a union - with or without children, with those sexually active outside marriage) will contribute to close a critical research gap and help accelerating the programmatic response for family planning provision for this group of women that is currently being left behind.

## Methods

We obtained data from nationally representative Demographic and Health Surveys (DHS) and Multiple Indicator Surveys (MICS) carried out since 2005 in LMICs. We used the most recently collected data for sexually active women aged 15–19 years from each country. DHS and MICS collect regular information on reproductive health and family planning using similar questionnaires, methodology and sampling strategy, which ensures the comparability of results. Both surveys employ multi-stage cluster sampling procedures to select women of reproductive age (15–49 years) for interview. The present analyses are focused on sexually active female adolescents, defined as 15–19 years-old women who are married or those who are not married but reported having had a sexual intercourse in the month preceding the interview. For convenience, we use the terms married/not married throughout the paper to refer to women formally married or those who reported being in a union (cohabiting with a partner), and the opposite.

The bulk of this study consists of a descriptive analysis of contraceptive use prevalence and demand for family planning satisfied coverage among female adolescents according to their marital status and number of children. For this purpose, adolescents were divided into three subgroups: not married sexually active, married without children, and married with children (at least one). We considered the number of children currently alive for each woman. Exceptions were Costa Rica, Panama, Suriname and Uzbekistan, for which the total number of children ever born (dead or alive) was considered due to the lack of detailed birth history information in the survey’s questionnaire.

In each survey, women were asked about current contraception use, including the type of contraceptive method, and about their current and future desire to have children. Two main indicators were analyzed in the present study: contraceptive use prevalence and DFPS. Contraceptive prevalence was measured as the percentage of sexually active women aged 15–19 years who report themselves or their partners as currently using at least one contraceptive method. DFPS was defined as the percentage of sexually active women 15–19 years currently using (or whose sexual partner is currently using) a contraceptive method to prevent pregnancy among those in need of contraception, being therefore a coverage indicator. Women in need of contraception are those who are sexually active, fecund and do not want to become pregnant within the next two years or are unsure whether or when they want to become pregnant. Pregnant women with a mistimed or unwanted pregnancy are also considered in need of contraception [16]. A detailed explanation of calculation models for the family planning indicators used can be found elsewhere [17]. We estimated contraceptive prevalence and DFPS with any contraceptive method (either modern or traditional) and for modern contraceptives methods only. The main analyses presented here are focused on modern contraceptive methods, considered the most effective ones. National estimates of contraceptive prevalence and DFPS for any method of contraception are presented in the Web Appendix.

In this work, we considered as traditional contraceptives the following methods: rhythm (calendar, standard days method, basal body temperature, symptothermal method, two-day method), withdrawal, lactation amenorrhea and “other traditional” methods. As modern contraceptives, we considered intrauterine devices and systems, subdermal implants, pills, injectable, diaphragms and cervical caps, condoms (male and female), spermicidal agents (foam, jelly, etc.), patch, emergency contraception, and sterilization (male and female).

Analytical methods included the estimation of median modern contraceptive prevalence and DFPS with modern methods (mDFPS) by country and world region for each subgroup of female adolescents (not married sexually active, married without children, married with children). World regions were considered according to the UNICEF classification: West & Central Africa, Eastern & Southern Africa, Middle East & North Africa, Europe & Central Asia, South Asia, East Asia & Pacific and Latin America & the Caribbean. Descriptive data to characterize the countries included their fertility rates (total and specifically for female adolescents), the prevalence of marriage before 18 years among women aged 20–24 years old and, the proportion of not married sexually active female adolescents in each country. Considering the complex sampling design of the surveys, all analyses took the clustering, sample weights and strata into account by using the command prefix svy. Stata 14 was used in the analyses (StataCorp. 2014. Stata Statistical Software: Release 14. College Station, TX: StataCorp LP).

## Results

We identified 81 countries with available information for sexually active female adolescents aged 15–19 years. For 8 of these countries we could not estimate the contraceptive prevalence and DFPS due to the limited sample sizes (*n* < 20 in each of the adolescent subgroups) in this age range: Armenia, Barbados, Belarus, Bosnia and Herzegovina, Kosovo, Montenegro, Santa Lucia, and Tajikistan. Thus, a total of 73 LMICs were included in this analysis (21 from West & Central Africa, 16 from Eastern & Southern Africa, 9 from Europe & Central Asia, 3 from South Asia, 8 from East Asia & the Pacific and 16 from Latin America & the Caribbean), of which 48 were DHS and 25 were MICS (Table 1).

**Table 1:** List of countries with available data on contraceptive use prevalence and DFPS among sexually active female adolescents and their characteristics

Region	Country	Year	Source	GDP	TFR	AFR	Early marriage	% sex active not married
West & Central Africa	Benin	2014	MICS	943	5.7	94	25.9	44.9
	Burkina Faso	2010	DHS	575	6	130	51.6	14.3
	Central African Republic	2010	MICS	446	6.2	229	67.9	10.0
	Cameroon	2014	MICS	1571	4.9	119	31	37.8
	Chad	2014	DHS	1025	6.4	179	66.9	7.5
	Congo Brazzaville	2011	DHS	3196	5.1	147	35.6	49.7
	Congo Democratic Republic	2013	DHS	458	6.6	138	37.3	31.4
	Cote d’Ivoire	2011	DHS	1214	5	129	33.2	46.8
	Gabon	2012	DHS	9774	4.1	114	21.9	57.7
	Gambia	2013	DHS	483	5.6	88	30.4	1.9
	Ghana	2014	DHS	1449	4.2	76	20.7	58.3
	Guinea	2012	DHS	665	5.1	146	51.7	16.1
	Guinea Bissau	2014	MICS	610	4.9	106	24.4	68.8
	Liberia	2013	DHS	454	4.7	149	35.9	58.6
	Mali	2015	MICS	749	6	151	49.7	11.8
	Niger	2012	DHS	391	7.6	206	76.3	0.3
	Nigeria	2016	MICS	2175	5.8	120	43.5	22.5
	Sao Tome and Principe	2014	MICS	1824	4.4	92	35.4	37.8
	Senegal	2016	DHS	952	4.9	80	31	2.3
	Sierra Leone	2013	DHS	710	4.9	125	38.9	56.8
	Togo	2013	DHS	579	4.8	84	21.8	45.3
Eastern & Southern Africa	Angola	2015	DHS	3683	6.2	163	30.3	43.9
	Burundi	2016	DHS	285	5.5	58	19	12.3
	Comoros	2012	DHS	788	4.3	70	31.6	5.2
	Ethiopia	2016	DHS	712	4.6	80	41.2	3.3
	Kenya	2014	DHS	1335	3.9	96	22.9	7.9
	Lesotho	2014	DHS	1218	3.3	94	17.3	17.7
	Madagascar	2008	DHS	470	4.8	148	48.1	15.4
	Malawi	2015	DHS	362	4.4	136	42.1	16.5
	Mozambique	2011	DHS	526	5.9	167	48.2	25.4
	Namibia	2013	DHS	5490	3.6	82	6.9	62.8
	Rwanda	2014	DHS	706	4.2	45	6.8	42.3
	Swaziland	2014	MICS	3379	3.3	87	5.3	59.0
	Tanzania	2015	DHS	872	5.2	132	30.5	19.1
	Uganda	2016	DHS	580	5.4	132	34	18.0
	Zambia	2013	DHS	1850	5.3	141	31.4	34.7
	Zimbabwe	2015	DHS	1033	4	110	32.4	10.6
Europe & Central Asia	Albania	2008	DHS	4370	1.6	17	9.6	18.9
	Azerbaijan	2006	DHS	5574	2	33	12.2	0.0
	Kazakhstan	2015	MICS	10,510	3	36	7	14.4
	Kyrgystan	2012	DHS	1177	3.6	44	7.8	0.6
	Macedonia	2005	MICS	4793	2.1	12	3.6	24.7
	Moldova	2012	MICS	2046	2.2	35	12.2	49.1
	Serbia	2010	MICS	5411	1.7	24	5	62.7
	Ukraine	2012	MICS	3855	1.5	34	9.1	47.5
	Uzbekistan	2006	MICS	1082	2.4	25	7	2.0
South Asia	Bhutan	2010	MICS	2178	2.6	59	25.8	2.6
	India	2015	DHS	1606	2.2	51	25.3	0.4
	Nepal	2016	DHS	729	2.3	88	39.5	0.1
East Asia & Pacific	Cambodia	2014	DHS	1093	2.7	57	18.5	1.1
	Indonesia	2012	DHS	3687	2.6	48	17	1.7
	Lao	2011	MICS	1381	3.2	94	35.4	3.9
	Mongolia	2013	MICS	4385	3.1	40	5.2	31.4
	Myanmar	2015	DHS	1138	2.3	36	16	0.0
	Philippines	2013	DHS	2760	3	57	15	9.9
	Timor Leste	2016	DHS	1987	4.2	42	14.9	3.8
	Vietnam	2010	MICS	1310	2	46	9.3	2.5
Latin America & Caribbean	Belize	2011	MICS	4517	2.6	64	25.9	26.3
	Bolivia	2008	DHS	1736	3.5	88	21.7	20.1
	Colombia	2015	DHS	6044	2	75	23.4	45.2
	Costa Rica	2011	MICS	9186	2.2	54	21.2	38.9
	Cuba	2014	MICS	7050	1.7	50	26	54.1
	Dominican Republic	2014	MICS	7195	2.5	89	35.9	30.1
	El Salvador	2014	MICS	3595	2.3	74	25.3	18.3
	Guatemala	2014	DHS	3687	3.1	92	29.5	9.8
	Guyana	2014	MICS	4030	2.6	74	30.2	9.0
	Haiti	2012	DHS	766	3.5	66	17.5	39.8
	Honduras	2011	DHS	2120	2.9	101	33.6	11.8
	Mexico	2015	MICS	9290	2.2	83	25.5	18.6
	Panama	2013	MICS	11,879	2.7	81	26.4	25.5
	Peru	2012	DHS	6387	2.6	64	19.1	30.3
	Suriname	2010	MICS	8303	2.5	66	18.8	53.9
	Trinidad and Tobago	2006	MICS	21,188	1.6	36	8.1	55.5

*TFR* total fertility rate; average number of children a woman would have by the end of her childbearing years (women age 15–49). *AFR* adolescent fertility rate; expressed per 1000 women aged 15–19. TFR and AFR are for the period 1–36 months preceding the survey. Early marriage: proportion of women aged 20–24 years whose first cohabitation was before 18 years. GDP per capita (current US$): World Bank national accounts data, and OECD National Accounts data files

Descriptive data regarding total fertility rates, adolescent birth rates, early marriage prevalence (women married before 18 years of age), as well as the proportion of not married sexually active adolescents are presented in Table 1 for each country included in the analyses. Adolescent birth rates above 100 births per 1000 adolescents were found in 24 countries, 23 of which from Africa (15 from West & Central Africa, 8 from Eastern & Southern Africa). Generally, a higher prevalence of early marriage was found in countries with higher adolescent birth rates. Central African Republic and Niger presented the highest adolescent birth rates and the highest prevalence of early marriage. In these countries, adolescent birth rates above 200 births per 1000 adolescents (229 per 1000 and 206 per 1000, respectively), and early marriage prevalence around 70% were observed (67.9 and 76.3%, respectively).

**Fig. 1:**
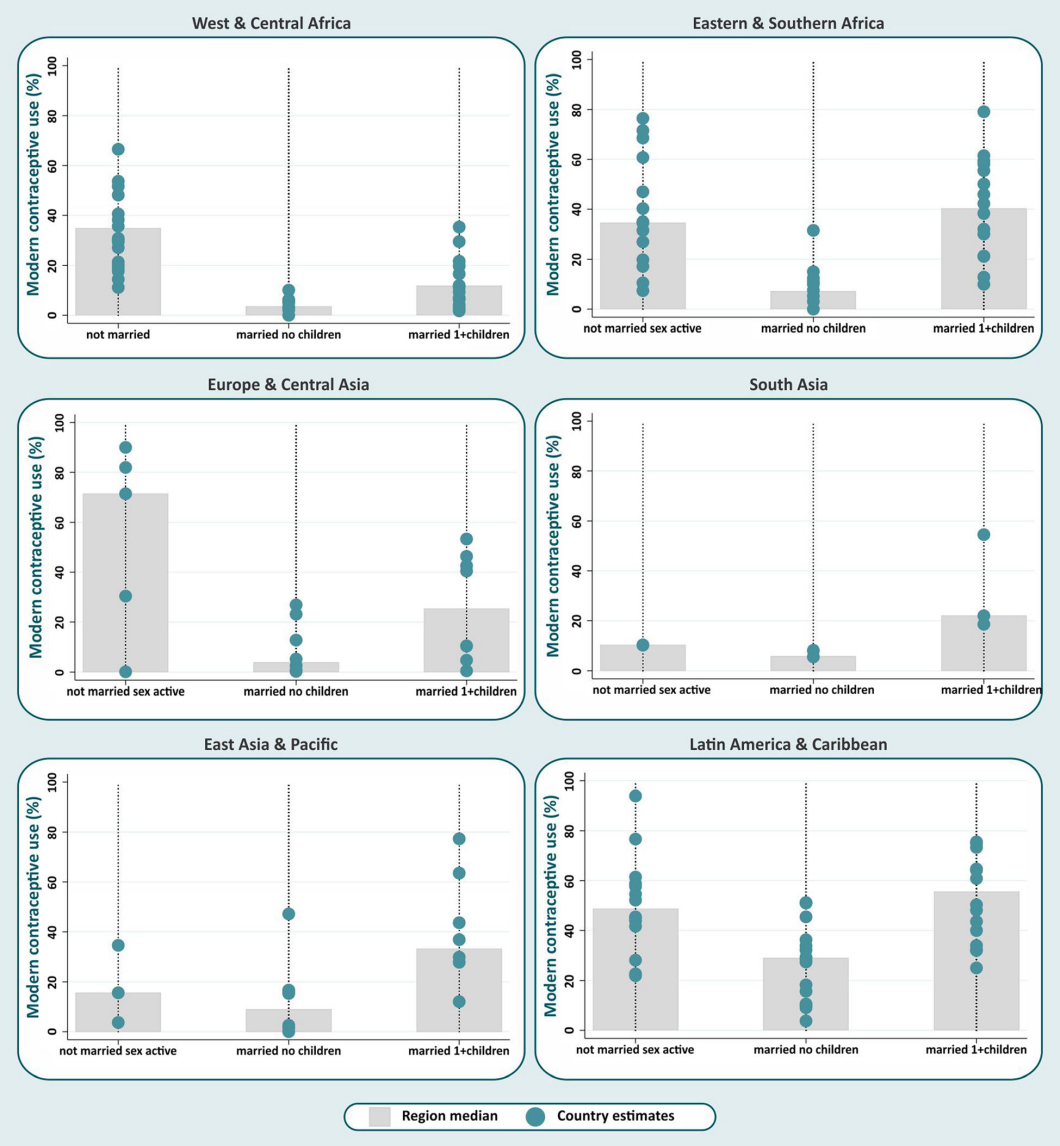
Median modern contraceptive use by female adolescent group and world region

**Fig. 2:**
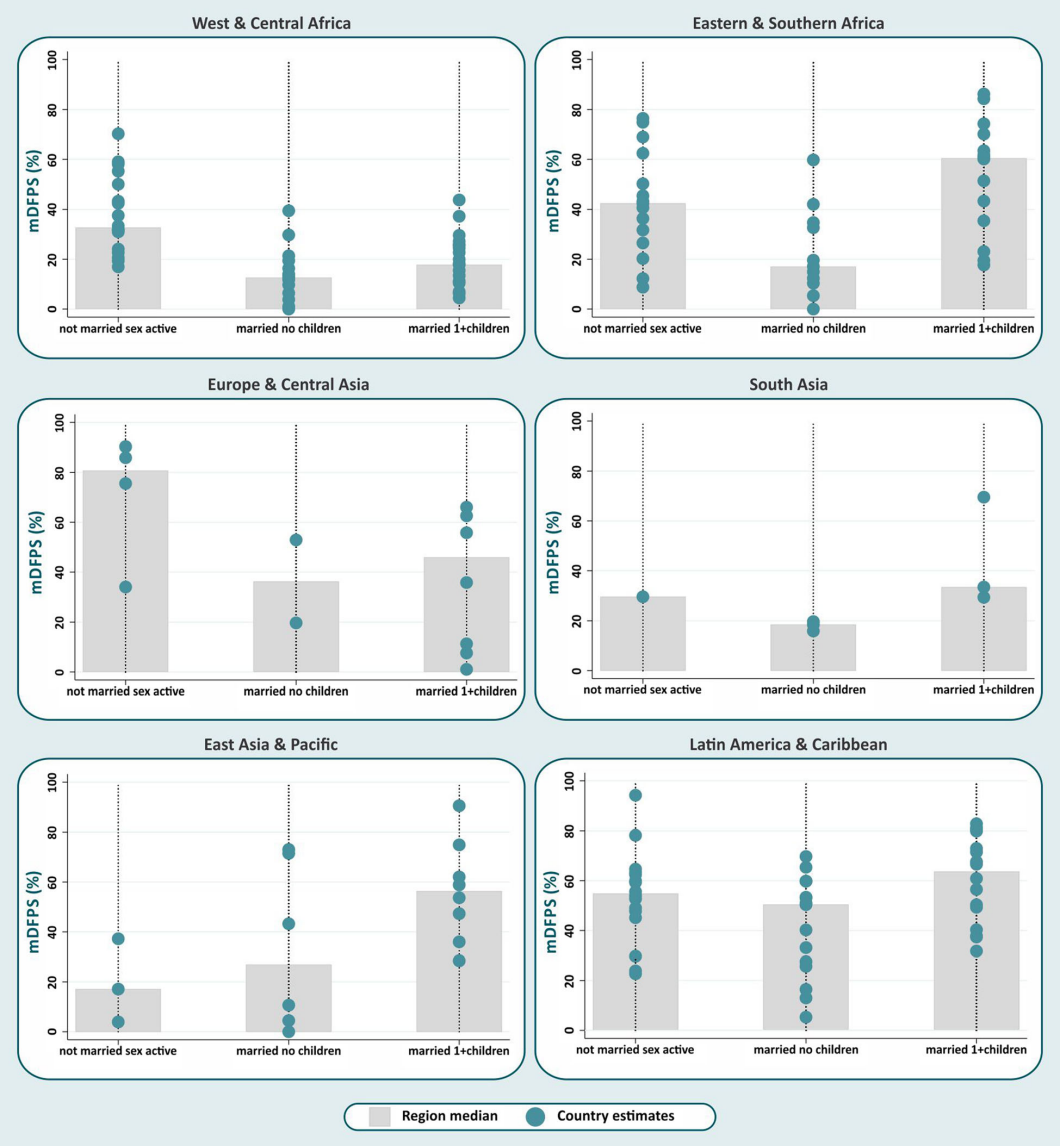
Median demand for family planning satisfied by modern methods (mDFPS) by female adolescent group and world region

Figures 1 and 2 show the medians for modern contraceptive prevalence and mDFPS among the distinct subgroups of female adolescents defined by their marital status and number of living children (not married sexually active, married without children, married with children).

The medians are represented by the grey bars, and individual country estimates are indicated by the blue dots. Expressive between-country inequalities in modern contraceptive prevalence and mDFPS coverage, revealed by the large spread of dots, were evident in all world regions. In general, similar patterns of distribution by adolescent groups were observed for the two indicators analyzed (Figs. 1 and 2). Married adolescents without children presented the lowest median modern contraceptive prevalence in all world regions, ranging from 2.9% in West & Central Africa to 29.0% in Latin America & Caribbean. The highest median modern contraceptive prevalence was observed for married adolescents with children, except for Europe & Central Asia and West & Central Africa, where not married sexually active adolescents were more likely to use modern contraception. For mDFPS, the same patterns were observed in most of the regions. West & Central Africa showed the lowest median mDFPS among married adolescents without children (12.6%), whereas Latin America & Caribbean presented the highest (50.4%). In East Asia & Pacific the lowest median mDFPS was among not married sexually active adolescents (17.1%).

As expected, mDFPS tended to be higher than modern contraceptive prevalence, as many female adolescents not using contraception reported a desire to become mothers. This was particularly evident among married adolescents without children. In Eastern & Southern Africa, for example, median modern contraceptive prevalence in this group was about 7.1%, while median mDFPS was 17.2%. Global and regional estimates of modern contraceptive prevalence and mDFPS by female adolescent group can also be found in Table 2.

**Table 2:** Median modern contraceptive prevalence and demand for family planning satisfied by modern methods (mDFPS) by adolescent group and world region

World region	Number of countries	Median modern contraceptive prevalence			Median mDFPS		
		Not married	Married no children	Married 1+ children	Not married	Married no children	Married 1+ children
West & Central Africa	21	30.5	2.9	9.2	32.7	12.6	17.8
Eastern & Southern Africa	16	34.6	7.1	40.3	42.4	17.2	60.5
Europe & Central Asia	9	71.4	3.9	25.4	80.7	36.3	45.9
South Asia	3	10.3	5.8	22.0	29.7	18.5	33.5
East Asia & Pacific	8	15.6	9.0	33.4	17.1	27.0	56.3
Latin America & Caribbean	16	48.8	29.0	55.6	54.8	50.4	63.7
Total	73	37.0	5.2	30.0	43.2	18.8	40.3

**Fig. 3:**
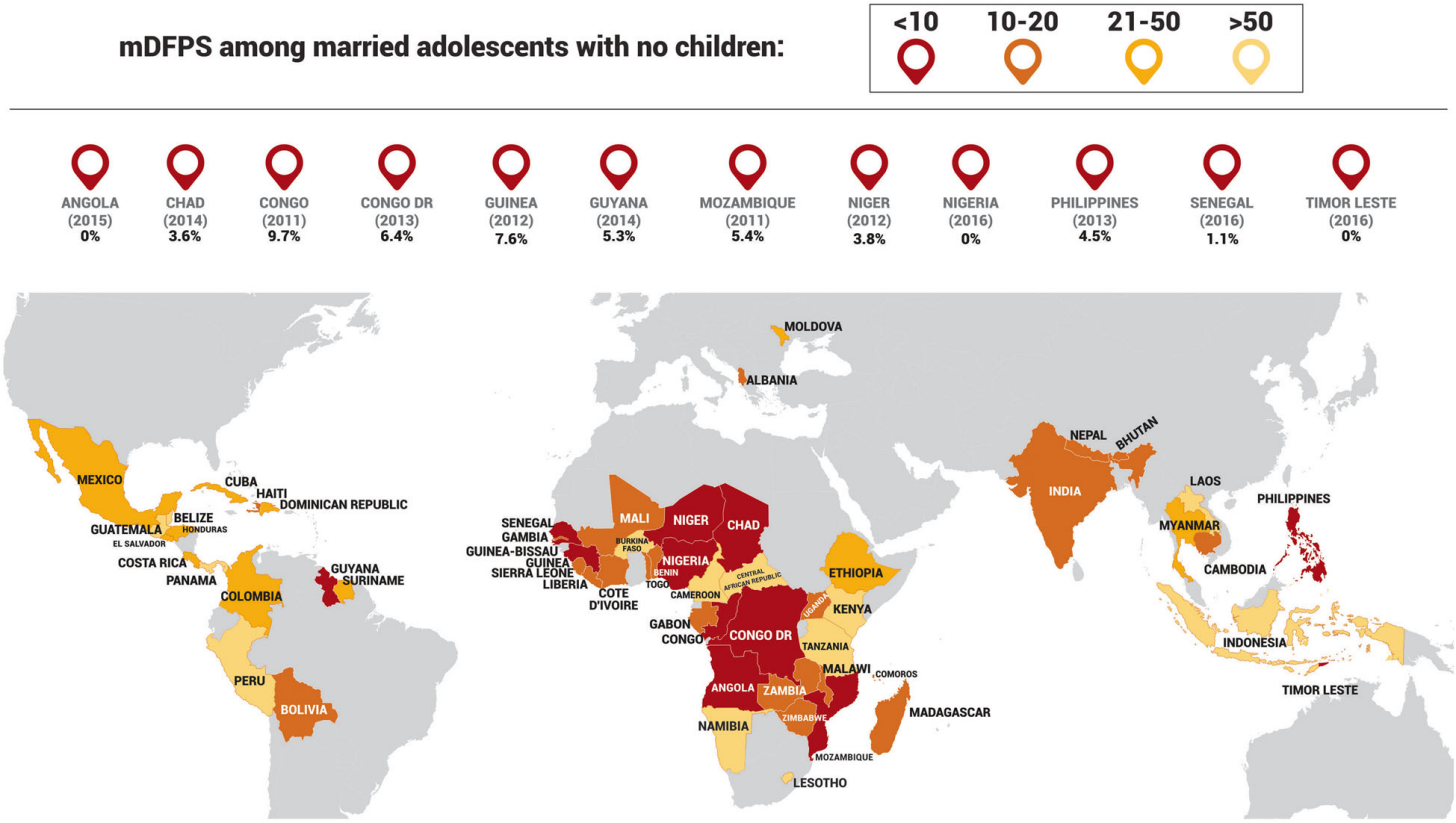
Demand for family planning satisfied by modern methods (mDFPS) coverage among married female adolescents with no children

In Fig. 3, mDFPS coverage among married female adolescents without children is presented for each of the countries in a world map. Countries where median mDFPS coverage was below 10% among married adolescents without children are highlighted at the top of the map. A total of 12 countries presented this scenario (Angola, Chad, Congo, Congo DR, Guinea, Guyana, Mozambique, Niger, Nigeria, Philippines, Senegal and, Timor Leste), 7 of which belong to West & Central Africa.

Individual country findings of modern contraceptive prevalence and mDFPS and a similar analysis for contraceptive prevalence and DFPS with any contraceptive method are presented in Additional files 1, 2, 3, 4 and 5. Overall, estimates were significantly higher with any methods as compared to modern methods in most West & Central Africa and Europe & Central Asia countries (particularly for married adolescents with children). In Europe & Central Asia, Albania presented the major differences between contraceptive use prevalence and DFPS with any (modern or traditional) and modern contraceptive methods only. For the other world regions, the contraceptive prevalence and DFPS estimates tend to be similar with those considering only modern methods. However, important differences (> 10 p.p. in at least one of the adolescent subgroups) between them were found for Peru, Honduras, Guatemala, Bolivia (Latin America & the Caribbean), Comoros, Madagascar, Tanzania (Eastern & Southern Africa), Cambodia, Philippines, Timor Leste, Vietnam, (East Asia & Pacific) and India (South Asia).

## Discussion

We present a broad overview of modern contraceptive prevalence and mDFPS coverage among female adolescents living in LMICs, considering their marital status and number of living children. Overall, we found that modern contraceptive prevalence and mDFPS were lower among adolescents who were married and had no children as compared to those married who had at least one child, or those sexually active outside marriage. No unique pattern was observed for female adolescents with the highest modern contraceptive prevalence and mDFPS coverage. While in Europe & Central Asia and West & Central Africa nations the highest modern contraceptive prevalence and mDFPS coverage were observed among not married sexually active adolescents, in all the other regions the highest estimates were found among married adolescents with children. Most relevant for policymaking, mDFPS among married adolescents without children was very low in all world regions – lowest in West & Central Africa, Eastern & Southern Africa, and in South Asia, where coverage was below 20%. Even in Latin America & the Caribbean where contraceptive use is generally higher [7], mDFPS coverage in this group was only 63.7%.

Our findings are consistent with previous research that have been conducted in some specific LMICs. In an analysis of household survey data from rural Southern Tanzania, Sedekia and colleagues [18] found that the unmet need for contraception was higher among women that wanted to delay the first birth compared to women who started childbearing and desired to wait at least two years before having another child (41% vs. 19%). Also in line with our findings, a study from Zimbabwe found that the proportion of adolescents with one or more children ever born that were currently using modern contraceptives was eight times higher than that of adolescents with no children (61% vs. 7.6%) [19]. Levels of contraceptive use before first pregnancy was also very low (7.9%) in India, even though the authors have found that the younger generation of married women are more likely to delay the start of childbearing as compared to their older counterparts [12].

Although marital status and parity have been identified as important individual characteristics influencing women’s reproductive health behaviors, including the uptake of modern contraception [20], the importance of community-based characteristics have been increasingly recognized to shape them [21]. Cultural values and gender norms strongly influence fertility desires and family planning needs, and in many places women are expected to give birth to at least one child before adopting contraception [11, 22, 23]. Restrictions by age and contraceptive method imposed by stakeholders in access and provision of modern contraceptives are also common challenges faced by women who wish to delay first birth [24]. In our study, even though modern contraceptive prevalence tend to be higher than mDFPS, reflecting the desire of some female adolescents who were not using contraception to become pregnant, mDFPS coverage is far below what would be desired, particularly in West & Central Africa and South Asia nations. These findings suggest a negative impact of cultural and social norms on contraceptive behavior of female adolescents.

A high proportion of early marriage was observed in many of the countries included in our study, particularly those from West & Central Africa. The association between early marriage and a lower likelihood of using contraceptives have already been pointed out in research findings from several countries where social norms tend to favor boys’ education and employment over girls’, and where young wives suffer and enormous pressure to bear children soon after marriage [12, 15, 25]. As a result, women regularly face pressure from their husbands, in-laws and others in the community not to use contraception [26, 27]. Besides, it is already known that marital relationships resulting from early marriage are more vulnerable to poor communication, sexual coercion and other forms of violence that results in low contraceptive use, putting women in a cycle of poverty with rapid and repeat childbearing [25, 28, 29].

The existing body of literature, considered in parallel with our own findings, strongly suggest that the goal of ensuring full access and choice to family planning and to improve maternal health will not be possible without tackling early marriage and prioritizing the reproductive health needs of married adolescents [15, 30]. In this sense, key programmatic objectives should be delay first births through social norm change and support female adolescents to space their subsequent births. Evidence of community-based multi-component approaches consisting of counseling and life-skills training of young married women, and their husbands, family and community members as well as capacity building of health workers have been showing promising effectiveness in increasing contraceptive use and delaying pregnancy in resource-constrained settings [31, 32]. Recent findings from a decade of the PRACHAR Project in India, showed that a gender-synchronized intervention tailored to specific life stages and based on a socioecological model approach was effective in sustain behavioral change efforts to voluntary increase contraceptive use among young married couples in a conservative area [33]. However, much of the existing evidence comes from small-scale research studies and projects with limitations in their methodology and evaluation design [1, 31]. There is also limited intervention programs on young married couples to delay first pregnancy as compared to spacing pregnancy. Further research is therefore needed to guide understanding of what can work to simultaneously reduce early marriage and the unmet need for family planning among married female adolescents. In this context, interventions grouping young women in line with their specific reproductive health needs (newly married, pregnant, mother of one/more children) have been found to be a successful innovative strategy to address the varying needs of different groups of female adolescents [1, 31].

We also point out from our findings that although not married sexually active women represent the minority of female adolescents and presented a higher modern contraceptive prevalence in some of the countries included in our study (particularly those from West & Central Africa and Europe & Central Asia), they should not be left behind in family planning initiatives [10]. Increased levels of contraception among sexually active unmarried female adolescents over time have been found to play an important role in fertility reductions that have been observed in many countries from Latin America and sub-Saharan Africa [34]. Besides, most of unmarried adolescents have a demand for family planning, which is usually larger than demand by married adolescents [34]. Even so, mDFPS coverage still lower among this group of female adolescents in most of the nations studied, reaching only 17% in East Asia & the Pacific. Commonly reported barriers to contraceptive use among not married sexually active adolescents include not being married and infrequent sex, both closely related to the taboo of pre-marital sex in these societies [35]. For the same reason, they remain a group that is often excluded from reproductive health surveys, services and strategies [10]. In this context, efforts focusing in supply-factors such as increasing access to contraception and providing adolescent-friendly services are key in responding to unmarried adolescent needs and not to leave any group aside.

Our analyses have some limitations. For some countries we couldn’t include the most recent survey because it didn’t collected information for sexually active women. This was the case for Kyrgystan, Macedonia, Nepal, Serbia and Vietnam. Also, Middle East & North Africa was not represented in our analysis because information for not married sexually active adolescents was not available in any of the surveys done in the region. However, our analyses covered 71% of the Family Planning 2020 priority countries for investment (49 out of 69 countries). Given the variability in the data collection period of surveys (2005–2016), caution is need when interpreting grouped findings and comparisons made between countries. Recent information is not available for many of the countries and we understand including surveys from 2005 onwards was a fair compromise between being inclusive and having recent data. In any case only seven countries have data from surveys before 2010. Also, the scenario in family planning has not changed dramatically over time [36], so that our results will be useful in terms of policy implications. Although previous studies had shown the relationship between contraception before first pregnancy and women’s education, wealth and area of residence, the limited sample sizes prevent us from describing contraception behavior of female adolescents according to these important dimensions of inequalities.

If not married female adolescents underreport sexual activity, this may have biased our estimate of mDFPS. If those adolescents that do not report sexual activity also do not use contraceptives as often as those who report, what is a likely scenario, our mDFPS estimates will tend to be overestimated. Underreporting of contraceptive use may also bias the estimates. But women who will not report her use of contraceptives due to social norms are unlikely to say they do not want a child soon. However, both our estimates of modern contraceptive use and mDFPS present low coverage. That would not happen if a relevant proportion of women were underreporting contraceptive use and the desire to postpone or not have children. In this case, mDFPS estimates would be considerably higher than the contraceptive use estimates.

Despite of these limitations, our study stands out as the first presenting a worldwide comprehensive analysis of contraceptive prevalence and DFPS among the distinct groups of female adolescents considering their marital status and number of children.

## Conclusions

Our findings indicate that most female adolescents who want to avoid pregnancy were not using a modern contraceptive method. Modern contraceptive prevalence and mDFPS were particularly low among married adolescents without children, which should be considered a priority group for intervention. Global efforts to prevent unintended pregnancies among female adolescents should consider contextual factors such as local social norms regarding marriage and fertility expectations so that family planning strategies can effectively reach adolescents everywhere. In this sense, interventions that engage both men and women and behavior change approaches tailored to adolescents have shown promising results to increase contraceptive use in more conservative contexts. These approaches should, therefore, be adapted to local contexts and aim at the priority groups highlighted in our study.

## Additional files

Additional file 1: Contraceptive use prevalence and demand for family planning satisfied coverage with any and modern methods among female adolescents in West & Central Africa countries. (DOCX 28 kb)

Additional file 2: Contraceptive use prevalence and demand for family planning satisfied coverage with any and modern methods among female adolescents in Eastern & Southern Africa countries. (DOCX 22 kb)

Additional file 3: Contraceptive use prevalence and demand for family planning satisfied coverage with any and modern methods among female adolescents in Europe & Central Asia countries. (DOCX 18 kb)

Additional file 4: Contraceptive use prevalence and demand for family planning satisfied coverage with any and modern methods among female adolescents in East Asia & Pacific, and South Asia countries. (DOCX 20 kb)

Additional file 5: Contraceptive use prevalence and demand for family planning satisfied coverage with any and modern methods among female adolescents in Latin America & Caribbean countries. (DOCX 24 kb)

## Abbreviations

DFPS: Demand for family planning satisfied
LMICs: Low- and middle-income countries
mDFPS: Demand for family planning satisfied with modern methods of contraception
