# Supplementary material for: Contraception in adolescence: the influence of parity and marital status on contraceptive use in 73 low-and middle-income countries
Source: Reprod Health. 2019 Feb 21;16:21. doi: 10.1186/s12978-019-0686-9 (PMC6383262; doi:10.1186/s12978-019-0686-9)
Supplement: Supplementary file 1 — Contraceptive use prevalence and demand for family planning satisfied coverage with any and modern methods among female adolescents in West & Central Africa countries. (DOCX 28 kb) [file 12978_2019_686_MOESM1_ESM.docx]

**Table A1. Contraceptive use prevalence and demand for family planning satisfied coverage with any and modern methods in West & Central Africa countries.**

| **Country** | **Source** | **Status** | **CPR**  **% (95%CI)** | **mCPR**  **% (95%CI)** | **N** | **DFPS**  **% (95%CI)** | **mDFPS**  **% (95%CI)** | **N** |
| --- | --- | --- | --- | --- | --- | --- | --- | --- |
| Benin (2014) | MICS | **Not married** | 21·5 (16·7-27·1) | 17·7 (13·3-23·2) | 376 | 23·4 (18·3-29·4) | 19·4 (14·6-25·2) | 343 |
|  |  | **Married no children** | 2·0 (0·5-6·9) | 2·0 (0·5-6·9) | 162 | 16·3 (4·7-43·9) | 16·3 (4·7-43·9) | 25 |
|  |  | **Married 1+ children** | 16·0 (11·0-22·8) | 9·2 (5·4-15·3) | 300 | 25·5 (17·5-35·6) | 15·4 (9·0-25·2) | 183 |
| Burkina Faso (2010) | DHS | **Not married** | 49·5 (39·6-59·4) | 48·1 (38·3-58·1) | 162 | 51·4 (41·1-61·6) | 50·0 (39·8-60·3) | 156 |
|  |  | **Married no children** | 6·5 (3·9-10·6) | 5·8 (3·3-10·0) | 486 | 43·8 (29·5-59·2) | 39·5 (25·6-55·3) | 59 |
|  |  | **Married 1+ children** | 6·7 (4·8-9·4) | 6·5 (4·6-9·1) | 484 | 16·0 (11·6-21·6) | 15·5 (11·2-21·1) | 217 |
| CAR (2010) | MICS | **Not married** | 14·4 (8·9-22·5) | 11·1 (6·3-19·0) | 149 | 24·8 (14·7-38·8) | 19·4 (11·0-31·7) | 85 |
|  |  | **Married no children** | 9·4 (6·0-14·4) | 6·3 (3·8-10·2) | 485 | 30·6 (20·9-42·3) | 21·4 (13·7-31·8) | 141 |
|  |  | **Married 1+ children** | 15·3 (12·2-19·2) | 7·0 (4·5-10·8) | 858 | 34·5 (28·3-41·2) | 17·8 (11·8-26·1) | 335 |
| Cameroon (2014) | MICS | **Not married** | 77·2 (71·0-82·4) | 66·5 (59·8-72·7) | 245 | 80·5 (74·4-85·5) | 70·3 (63·5-76·3) | 234 |
|  |  | **Married no children** | 8·6 (4·5-15·9) | 4·7 (1·8-11·3) | 144 | 55·1 (31·5-76·6) | 29·8 (12·1-56·7) | 24 |
|  |  | **Married 1+ children** | 32·9 (26·3-40·3) | 12·0 (8·2-17·1) | 260 | 63·2 (53·7-71·8) | 29·1 (21·4-39·3) | 114 |
| Chad (2014) | DHS | **Not married** | 15·8 (9·5-25·3) | 14·4 (8·3-24·0) | 123 | 18·6 (11·1-29·4) | 16·9 (9·7-27·9) | 107 |
|  |  | **Married no children** | 0·7 (0·2-2·3) | 0·7 (0·2-2·3) | 540 | 3·6 (1·1-11·1) | 3·6 (1·1-11·1) | 103 |
|  |  | **Married 1+ children** | 4·0 (2·4-6·8) | 1·8 (1·0-3·1) | 976 | 14·3 (8·9-22·1) | 6·3 (3·6-10·6) | 257 |
| Congo (2011) | DHS | **Not married** | 74·3 (68·6-79·3) | 40·6 (34·0-47·5) | 466 | 79·3 (73·4-84·2) | 43·3 (36·6-50·2) | 428 |
|  |  | **Married no children** | 8·2 (3·6-17·5) | 4·5 (1·3-14·8) | 112 | 17·6 (7·5-36·2) | 9·7 (2·7-29·6) | 49 |
|  |  | **Married 1+ children** | 45·8 (39·1-52·6) | 29·6 (23·4-36·6) | 327 | 57·7 (49·6-65·3) | 37·3 (30·3-44·8) | 252 |
| Congo DR (2013) | DHS | **Not married** | 44·5 (36·4-53·0) | 19·1 (13·7-25·9) | 454 | 48·1 (39·5-56·9) | 20·6 (14·8-27·9) | 411 |
|  |  | **Married no children** | 5·5 (2·9-10·3) | 1·7 (0·7-4·0) | 383 | 20·9 (11·5-35·0) | 6·4 (2·7-14·6) | 94 |
|  |  | **Married 1+ children** | 16·7 (13·1-21·2) | 7·2 (4·7-10·8) | 583 | 31·3 (24·7-38·7) | 13·5 (8·8-20·1) | 295 |
| Cote d´Ivoire (2011) | DHS | **Not married** | 35·2 (29·4-41·5) | 29·5 (24·1-35·5) | 369 | 37·9 (31·6-44·7) | 31·7 (25·9-38·2) | 334 |
|  |  | **Married no children** | 6·7 (3·2-13·8) | 2·5 (0·9-6·6) | 184 | 36·9 (18·4-60·3) | 13·5 (4·9-32·1) | 35 |
|  |  | **Married 1+ children** | 14·5 (9·5-21·4) | 9·7 (5·8-15·8) | 228 | 27·0 (17·8-38·8) | 18·2 (11·0-28·6) | 113 |
| Gabon (2012) | DHS | **Not married** | 61·7 (50·5-71·8) | 51·7 (41·4-61·8) | 408 | 66·0 (54·0-76·2) | 55·3 (44·3-65·8) | 377 |
|  |  | **Married no children** | 23·2 (12·6-38·9) | 10·1 (5·3-18·2) | 103 | 44·8 (25·7-65·7) | 19·4 (10·1-34·2) | 53 |
|  |  | **Married 1+ children** | 26·2 (16·7-38·8) | 19·8 (11·5-31·7) | 183 | 33·9 (22·0-48·2) | 25·5 (15·2-39·6) | 138 |
| Gambia (2013) | DHS | **Not married** | --- | --- | 12 | --- | --- | 10 |
|  |  | **Married no children** | 1·5 (0·3-8·3) | 1·5 (0·3-8·3) | 311 | 11·8 (2·0-46·0) | 11·8 (2·0-46·0) | 39 |
|  |  | **Married 1+ children** | 5·2 (2·5-10·4) | 2·9 (1·4-6·0) | 303 | 18·6 (9·4-33·7) | 10·5 (5·1-20·1) | 91 |
| Ghana (2014) | DHS | **Not married** | 42·9 (33·6-52·8) | 30·1 (21·7-40·1) | 175 | 45·6 (35·7-55·9) | 32·0 (23·0-42·6) | 166 |
|  |  | **Married no children** | 8·4 (2·0-28·9) | 3·3 (0·5-20·3) | 37 | --- | --- | 14 |
|  |  | **Married 1+ children** | 22·4 (13·1-35·5) | 21·7 (12·6-34·8) | 85 | 28·0 (16·2-44·0) | 27·2 (15·5-43·2) | 67 |
| Guinea (2012) | DHS | **Not married** | 26·2 (19·1-34·8) | 21·4 (15·1-29·4) | 128 | 29·5 (21·8-38·4) | 24·1 (17·3-32·5) | 118 |
|  |  | **Married no children** | 0·7 (0·2-2·5) | 0·7 (0·2-2·5) | 260 | 7·6 (2·0-25·4) | 7·6 (2·0-25·4) | 26 |
|  |  | **Married 1+ children** | 4·2 (2·5-7·1) | 1·7 (0·7-4·1) | 407 | 11·1 (6·5-18·3) | 4·5 (1·8-10·6) | 153 |
| Guinea Bissau (2014) | MICS | **Not married** | 58·4 (53·1-63·6) | 53·7 (48·7-58·7) | 622 | 63·5 (58·1-68·9) | 59·0 (53·7-64·2) | 558 |
|  |  | **Married no children** | 4·0 (1·4-10·7) | 4·0 (1·4-10·7) | 94 | --- | --- | 17 |
|  |  | **Married 1+ children** | 9·4 (5·2-16·5) | 4·2 (1·6-10·7) | 188 | 22·8 (12·6-37·7) | 11·2 (4·2-26·5) | 65 |
| Liberia (2013) | DHS | **Not married** | 32·8 (27·0-39·1) | 30·9 (25·7-36·5) | 514 | 35·5 (29·2-42·3) | 33·4 (27·7-39·5) | 462 |
|  |  | **Married no children** | 6·0 (1·4-21·8) | 6·0 (1·4-21·8) | 101 | 14·0 (3·1-44·7) | 14·0 (3·1-44·7) | 40 |
|  |  | **Married 1+ children** | 16·6 (10·2-25·9) | 16·6 (10·2-25·9) | 251 | 24·5 (15·5-36·5) | 24·5 (15·5-36·5) | 173 |
| Mali (2015) | MICS | **Not married** | 27·8 (21·5-35·0) | 27·1 (20·9-34·4) | 197 | 31·7 (24·5-39·9) | 31·0 (23·9-39·1) | 179 |
|  |  | **Married no children** | 3·2 (2·0-5·1) | 2·9 (1·7-4·7) | 634 | 15·6 (10·0-23·6) | 14·0 (8·7-21·7) | 136 |
|  |  | **Married 1+ children** | 11·7 (9·3-14·7) | 11·2 (8·8-14·2) | 838 | 26·9 (21·7-32·8) | 25·9 (20·8-31·8) | 354 |
| Niger (2012) | DHS | **Not married** | --- | --- | --- | --- | --- | 2 |
|  |  | **Married no children** | 0·7 (0·3-1·8) | 0·4 (0·1-1·5) | 496 | 6·5 (2·5-15·6) | 3·8 (1·1-12·9) | 57 |
|  |  | **Married 1+ children** | 13·4 (10·1-17·4) | 5·8 (4·0-8·5) | 509 | 45·5 (37·1-54·2) | 19·9 (13·8-27·9) | 166 |
| Nigeria (2016) | DHS | **Not married** | 24·2 (18·4-31·1) | 20·1 (14·7-26·9) | 262 | 27·0 (20·8-34·2) | 22·7 (16·8-29·9) | 236 |
|  |  | **Married no children** | 0 | 0 | 560 | 0 | 0 | 70 |
|  |  | **Married 1+ children** | 3·6 (2·5-5·3) | 2·1 (1·3-3·5) | 827 | 11·6 (7·9-16·8) | 7·0 (4·2-11·5) | 265 |
| Sao Tome & Principe (2014) | MICS | **Not married** | 41·2 (26·5-57·5) | 38·3 (24·0-55·0) | 68 | 45·6 (29·6-62·6) | 42·5 (26·8-60·0) | 61 |
|  |  | **Married no children** | 2·1 (0·3-14·3) | 2·1 (0·3-14·3) | 27 | --- | --- | 11 |
|  |  | **Married 1+ children** | 38·5 (26·5-52·1) | 35·4 (24·1-48·6) | 85 | 47·6 (33·3-62·4) | 43·8 (30·4-58·2) | 69 |
| Senegal (2016) | DHS | **Not married** | --- | --- | 14 | --- | --- | 13 |
|  |  | **Married no children** | 0·4 (0·1-1·7) | 0·2 (0·0-1·7) | 319 | 1·9 (0·5-7·8) | 1·1 (0·1-7·7) | 81 |
|  |  | **Married 1+ children** | 14·0 (9·5-20·2) | 10·3 (6·6-15·8) | 281 | 30·8 (20·7-43·1) | 22·7 (14·7-33·3) | 115 |
| Sierra Leone (2013) | DHS | **Not married** | 56·4 (51·1-61·5) | 53·7 (48·6-58·7) | 974 | 61·0 (55·6-66·2) | 58·1 (52·9-63·2) | 907 |
|  |  | **Married no children** | 5·1 (3·1-8·4) | 5·1 (3·1-8·4) | 302 | 19·1 (11·2-30·4) | 19·1 (11·2-30·4) | 77 |
|  |  | **Married 1+ children** | 9·6 (6·8-13·5) | 6·3 (4·2-9·4) | 433 | 20·6 (14·9-27·7) | 13·6 (9·2-19·6) | 201 |
| Togo (2013) | DHS | **Not married** | 41·7 (33·4-50·4) | 35·7 (28·7-43·5) | 199 | 43·8 (35·3-52·7) | 37·6 (30·3-45·5) | 190 |
|  |  | **Married no children** | 3·4 (0·7-14·2) | 3·4 (0·7-14·2) | 80 | 12·6 (2·9-41·2) | 12·6 (2·9-41·2) | 21 |
|  |  | **Married 1+ children** | 10·8 (6·6-17·3) | 9·4 (5·4-15·7) | 159 | 17·6 (10·7-27·6) | 15·2 (8·8-24·9) | 105 |

--- not enough sample size; n<20
