# Supplementary material for: Contraception in adolescence: the influence of parity and marital status on contraceptive use in 73 low-and middle-income countries
Source: Reprod Health. 2019 Feb 21;16:21. doi: 10.1186/s12978-019-0686-9 (PMC6383262; doi:10.1186/s12978-019-0686-9)
Supplement: Supplementary file 2 — Contraceptive use prevalence and demand for family planning satisfied coverage with any and modern methods among female adolescents in Eastern & Southern Africa countries. (DOCX 22 kb) [file 12978_2019_686_MOESM2_ESM.docx]

**Additional file 2. Contraceptive use prevalence and demand for family planning satisfied coverage with any and modern methods in Eastern & Southern Africa countries.**

| **Country** | **Source** | **Status** | **CPR**  **% (95%CI)** | **mCPR**  **% (95%CI)** | **N** | **DFPS**  **% (95%CI)** | **mDFPS**  **% (95%CI)** | **N** |
| --- | --- | --- | --- | --- | --- | --- | --- | --- |
| Angola (2015) | DHS | **Not married** | 21·1 (16·7-26·3) | 19·8 (15·5-24·9) | 547 | 28·3 (22·6-34·7) | 26·5 (21·0-33·0) | 419 |
|  |  | **Married no child** | 0 | 0 | 158 | 0 | 0 | 47 |
|  |  | **Married 1+ child** | 10·7 (7·3-15·4) | 10·1 (6·9-14·9) | 528 | 18·6 (12·8-26·3) | 17·7 (12·0-25·3) | 278 |
| Burundi (2016) | DHS | **Not married** | 37·2 (19·5-59·0) | 34·6 (17·6-56·8) | 29 | 46·3 (23·9-70·3) | 43·2 (21·6-67·7) | 23 |
|  |  | **Married no child** | 0.6 (0.1-4.4) | 0 | 86 | --- | --- | 8 |
|  |  | **Married 1+ child** | 43·3 (33·2-54·0) | 38·4 (28·9-48·8) | 121 | 68·5 (55·6-79·0) | 60·7 (48·0-72·0) | 74 |
| Comoros (2012) | DHS | **Not married** | --- | --- | 13 | --- | --- | 13 |
|  |  | **Married no child** | 19·5 (13·0-28·1) | 11·0 (6·2-18·9) | 125 | 28·4 (19·2-39·9) | 16·1 (8·8-27·7) | 89 |
|  |  | **Married 1+ child** | 19·8 (12·6-29·8) | 12·2 (7·2-21·9) | 107 | 30·1 (19·2-43·7) | 19·5 (11·0-32·2) | 65 |
| Ethiopia (2016) | DHS | **Not married** | 63·9 (33·8-85·9) | 60·8 (31·7-83·8) | 23 | 65·6 (34·2-87·5) | 62·4 (32·1-85·4) | 22 |
|  |  | **Married no child** | 31·6 (24·5-39·7) | 31·6 (24·5-39·7) | 370 | 59·8 (47·0-71·4) | 59·8 (47·0-71·4) | 165 |
|  |  | **Married 1+ child** | 32·3 (24·8-40·9) | 32·1 (24·6-40·7) | 294 | 62·1 (51·7-71·5) | 61·7 (51·2-71·2) | 145 |
| Kenya  (2014) | DHS | **Not married** | 47·8 (32·0-64·1) | 47·0 (31·3-63·4) | 68 | 51·1 (34·3-67·6) | 50·2 (33·5-66·9) | 63 |
|  |  | **Married no child** | 11·6 (6·5-19·9) | 10·1 (5·2-18·5) | 274 | 46·7 (27·1-67·4) | 42·1 (23·1-63·7) | 37 |
|  |  | **Married 1+ child** | 54·4 (48·9-59·8) | 50·1 (44·3-55·9) | 514 | 66·9 (57·7-75·0) | 60·1 (50·4-69·2) | 152 |
| Lesotho (2014) | DHS | **Not married** | 72·2 (59·1-82·3) | 68·6 (55·0-79·6) | 58 | 72·6 (59·4-82·7) | 69·0 (55·2-80·0) | 57 |
|  |  | **Married no child** | 12·4 (7·5-19·8) | 12·4 (7·5-19·8) | 130 | 32·8 (21·4-46·8) | 32·8 (21·4-46·8) | 47 |
|  |  | **Married 1+ child** | 55·5 (45·0-65·5) | 55·5 (45·0-65·5) | 137 | 63·5 (52·5-73·2) | 63·5 (52·5-73·2) | 116 |
| Madagascar (2008) | DHS | **Not married** | 31·9 (25·4-39·3) | 10·5 (6·8-15·9) | 258 | 37·1 (29·6-45·4) | 12·2 (8·0-18·4) | 221 |
|  |  | **Married no child** | 19·8 (16·2-24·0) | 7·6 (5·3-10·7) | 632 | 39·0 (32·6-45·9) | 15·0 (10·8-20·5) | 308 |
|  |  | **Married 1+ child** | 28·6 (24·3-33·4) | 21·1 (17·4-25·4) | 734 | 54·9 (48·7-60·9) | 40·5 (34·6-46·8) | 391 |
| Malawi (2015) | DHS | **Not married** | 33·5 (26·1-41·8) | 31·6 (24·5-39·6) | 242 | 38·5 (30·6-47·1) | 36·4 (28·7-44·8) | 216 |
|  |  | **Married no child** | 2·9 (1·7-4·8) | 2·9 (1·7-4·8) | 467 | 10·3 (6·3-16·6) | 10·3 (6·3-16·6) | 138 |
|  |  | **Married 1+ child** | 62·5 (57·7-67·0) | 61·5 (56·7-66·0) | 732 | 75·5 (71·0-79·4) | 74·3 (69·8-78·3) | 619 |
| Mozambique (2011) | DHS | **Not married** | 27·2 (21·7-33·5) | 27·0 (21·5-33·3) | 374 | 32·0 (25·6-39·1) | 31·7 (25·4-38·8) | 322 |
|  |  | **Married no child** | 0·8 (0·4-1·6) | 0·7 (0·3-1·5) | 487 | 6·1 (2·9-12·4) | 5·4 (2·4-11·5) | 72 |
|  |  | **Married 1+ child** | 10·3 (7·8-13·7) | 10·0 (7·4-13·3) | 585 | 24·0 (18·1-31·0) | 23·1 (17·3-30·1) | 266 |
| Namibia (2013) | DHS | **Not married** | 71·8 (64·7-78·0) | 71·6 (64·5-77·8) | 196 | 75·2 (67·9-81·2) | 74·9 (67·6-81·0) | 185 |
|  |  | **Married no child** | 15·0 (6·7-30·3) | 15·0 (6·7-30·3) | 39 | 32·7 (15·0-57·1) | 32·7 (15·0-57·1) | 23 |
|  |  | **Married 1+ child** | 50·2 (35·6-64·7) | 42·3 (28·8-56·9) | 73 | 61·0 (44·1-75·6) | 51·4 (35·1-67·3) | 62 |
| Rwanda (2014) | DHS | **Not married** | 7·4 (2·8-18·0) | 7·4 (2·8-18·0) | 63 | 8·8 (3·4-20·9) | 8·8 (3·4-20·9) | 54 |
|  |  | **Married no child** | 0 | 0 | 35 | --- | --- | 0 |
|  |  | **Married 1+ child** | 63·9 (47·2-77·7) | 59·4 (43·5-73·5) | 46 | 90·7 (72·4-97·3) | 84·3 (65·6-93·8) | 33 |
| Swaziland (2014) | MICS | **Not married** | 76·5 (62·6-86·3) | 76·5 (62·6-86·3) | 59 | 76·5 (62·6-86·3) | 76·5 (62·6-86·3) | 59 |
|  |  | **Married no child** | --- | --- | 14 | --- | --- | 7 |
|  |  | **Married 1+ child** | 58·1 (37·2-76·5) | 58·1 (37·2-76·5) | 27 | 70·2 (48·7-85·3) | 70·2 (48·7-85·3) | 24 |
| Tanzania (2015) | DHS | **Not married** | 41·9 (33·3-51·1) | 35·0 (27·0=44·1) | 151 | 48·7 (39·7-57·9) | 40·7 (31·7-50·4) | 133 |
|  |  | **Married no child** | 4·1 (2·1-7·7) | 3·2 (1·5-6·4) | 267 | 45·1 (25·2-66·8) | 34·7 (17·3-57·4) | 25 |
|  |  | **Married 1+ child** | 23·0 (18·5-28·2) | 21·3 (16·9-26·5) | 352 | 38·3 (31·5-45·5) | 35·4 (28·8-42·7) | 206 |
| Uganda (2016) | DHS | **Not married** | 42·8 (34·8-51·2) | 40·3 (32·3-48·8) | 192 | 48·3 (39·3-57·5) | 45·4 (25·5-58·3) | 166 |
|  |  | **Married no child** | 6·2 (3·7-10·1) | 5·2 (3·0-9·1) | 351 | 23·1 (14·6-34·6) | 19·6 (11·6-31·3) | 95 |
|  |  | **Married 1+ child** | 32·5 (27·8-37·5) | 30·1 (25·6-35·1) | 524 | 46·7 (40·5-53·0) | 43·4 (37.3-49·6) | 359 |
| Zambia (2013) | DHS | **Not married** | 17·8 (13·0-23·9) | 17·1 (12·3-23·3) | 310 | 21·0 (15·5-27·8) | 20·3 (14·7-27·3) | 269 |
|  |  | **Married no child** | 3·1 (1·1-8·9) | 3·1 (1·1-8·9) | 163 | 12·3 (4·2-30·9) | 12·3 (4·2-30·9) | 44 |
|  |  | **Married 1+ child** | 49·8 (44·0-55·6) | 46·0 (40·3-51·8) | 409 | 65·6 (59·2-71·4) | 60·5 (54·0-66·7) | 311 |
| Zimbabwe (2015) | DHS | **Not married** | 33·6 (20·4-50·1) | 33·6 (20·4-50·1) | 46 | 42·4 (26·4-60·3) | 42·4 (26·4-60·3) | 39 |
|  |  | **Married no child** | 3·6 (1·6-8·0) | 3·0 (1·2-7·2) | 162 | 20·5 (9·0-40·3) | 17·1 (6·9-36·5) | 32 |
|  |  | **Married 1+ child** | 80·3 (74·2-85·2) | 79·1 (72·9-84·2) | 219 | 87·5 (81·8-91·6) | 86·2 (80·3-90·6) | 202 |

--- not enough sample size; n<20
