# Supplementary material for: Contraception in adolescence: the influence of parity and marital status on contraceptive use in 73 low-and middle-income countries
Source: Reprod Health. 2019 Feb 21;16:21. doi: 10.1186/s12978-019-0686-9 (PMC6383262; doi:10.1186/s12978-019-0686-9)
Supplement: Supplementary file 3 — Contraceptive use prevalence and demand for family planning satisfied coverage with any and modern methods among female adolescents in Europe & Central Asia countries. (DOCX 18 kb) [file 12978_2019_686_MOESM3_ESM.docx]

**Additional file 3. Contraceptive use prevalence and demand for family planning satisfied coverage with any and modern methods in Europe & Central Asia countries.**

| **Country** | **Source** | **Status** | **CPR**  **% (95%CI)** | **mCPR**  **% (95%CI)** | **N** | **DFPS**  **% (95%CI)** | **mDFPS**  **% (95%CI)** | **N** |
| --- | --- | --- | --- | --- | --- | --- | --- | --- |
| Albania (2008) | DHS | **Not married** | 79·5 (49·5-93·9) | 30·4 (13·6-54·9) | 24 | 89·2 (62·6-97·6) | 34·1 (15·3-59·7) | 23 |
|  |  | **Married no child** | 54·6 (38·9-69·4) | 12·8 (5·1-28·3) | 76 | 84·4 (68·2-93·2) | 19·7 (7·9-41·2) | 48 |
|  |  | **Married 1+ child** | 54·9 (35·3-73·1) | 10·4 (2·4-35·4) | 26 | 59·5 (37·8-78·0) | 11·3 (2·5-39·0) | 22 |
| Azerbaijan (2006) | DHS | **Not married** | --- | --- | 0 | --- | --- | 0 |
|  |  | **Married no child** | 2·5 (0·3-17·3) | 2·5 (0·3-17·3) | 97 | --- | --- | 9 |
|  |  | **Married 1+ child** | 14·0 (5·9-29·8) | 0·5 (0·1-4·0) | 54 | 28·7 (4·5-77·5) | 1·0 (0·0-46·8) | 30 |
| Kazakhstan (2015) | MICS | **Not married** | --- | --- | 14 | --- | --- | 13 |
|  |  | **Married no child** | 6·8 (2·4-18·1) | 5·2 (1·6-16·3) | 42 | --- | --- | 6 |
|  |  | **Married 1+ child** | 48·8 (32·2-65·7) | 40·5 (25·2-57·8) | 41 | 72·2 (50·4-86·9) | 66·1 (43·4-83·2) | 27 |
| Kyrgystan (2012) | DHS | **Not married** | --- | --- | 1 | --- | --- | 1 |
|  |  | **Married no child** | 0·8 (0·1-5·5) | 0·8 (0·1-5·5) | 98 | --- | --- | 7 |
|  |  | **Married 1+ child** | 12·3 (6·3-22·6) | 10·3 (4·8-21·0) | 66 | 42·7 (21·6-66·8) | 35·9 (15·6-63·0) | 23 |
| Macedonia (2005) | MICS | **Not married** | 0·1 (0·0-0·9) | 0·1 (0·0-0·9) | 39 | --- | --- | 1 |
|  |  | **Married no child** | 0·3 (0·0-2·4) | 0·3 (0·0-2·4) | 33 | --- | --- | 3 |
|  |  | **Married 1+ child** | 5·5 (1·9-15·0) | 4·8 (1·4-14·9) | 86 | 8·7 (2·7-24·9) | 7·6 (2·0-24·3) | 41 |
| Moldova (2012) | MICS | **Not married** | 73·4 (62·0-82·4) | 71·4 (59·9-80·6) | 79 | 77·7 (65·9-86·3) | 75·6 (63·6-84·5) | 74 |
|  |  | **Married no child** | 33·5 (19·9-50·5) | 26·9 (15·1-43·1) | 47 | 61·4 (37·5-80·8) | 52·9 (31·2-73·6) | 27 |
|  |  | **Married 1+ child** | 59·4 (42·1-74·6) | 46·4 (30·0-63·5) | 35 | 69·4 (50·5-83·4) | 55·8 (36·9-73·2) | 30 |
| Serbia (2010) | MICS | **Not married** | 93·7 (82·0-98·0) | 90·0 (76·6-96·1) | 64 | 93·7 (81·7-98·0) | 90·3 (76·-96·4) | 63 |
|  |  | **Married no child** | --- | --- | 7 | --- | --- | 1 |
|  |  | **Married 1+ child** | 46·3 (20·7-74·0) | 1·8 (0·2-13·7) | 31 | --- | --- | 16 |
| Ukraine (2012) | MICS | **Not married** | 85·8 (68·5-94·3) | 81·9 (65·5-91·5) | 58 | 89·9 (74·9-96·4) | 85·9 (71·6-93·6) | 57 |
|  |  | **Married no child** | --- | --- | 16 | --- | --- | 9 |
|  |  | **Married 1+ child** | 63·8 (42·8-80·7) | 42·6 (22·8-65·1) | 48 | 89·3 (75·4-95·8) | 62·7 (36·7-83·1) | 37 |
| Uzbekistan (2006) | MICS | **Not married** | --- | --- | 3 | --- | --- | 3 |
|  |  | **Married no child** | 2·6 (0·9-7·3) | 2·6 (0·9-7·3) | 104 | --- | --- | 9 |
|  |  | **Married 1+ child** | 64·6 (48·6-77·9) | 53·3 (36·8-69·2) | 46 | 75·8 (59·2-87·1) | 62·6 (44·2-78·0) | 38 |

--- not enough sample size; n<20.
