# Supplementary material for: Contraception in adolescence: the influence of parity and marital status on contraceptive use in 73 low-and middle-income countries
Source: Reprod Health. 2019 Feb 21;16:21. doi: 10.1186/s12978-019-0686-9 (PMC6383262; doi:10.1186/s12978-019-0686-9)
Supplement: Supplementary file 4 — Contraceptive use prevalence and demand for family planning satisfied coverage with any and modern methods among female adolescents in East Asia & Pacific, and South Asia countries. (DOCX 20 kb) [file 12978_2019_686_MOESM4_ESM.docx]

**Additional file 4. Contraceptive use prevalence and demand for family planning satisfied coverage with any and modern methods in East Asia & Pacific, and South Asia countries.**

| **Country** | **Source** | **Status** | **CPR**  **% (95%CI)** | **mCPR**  **% (95%CI)** | **N** | **DFPS**  **% (95%CI)** | **mDFPS**  **% (95%CI)** | **N** |
| --- | --- | --- | --- | --- | --- | --- | --- | --- |
| Cambodia (2014) | DHS | **Not married** | --- | --- | 5 | --- | --- | 5 |
|  |  | **Married no children** | 13·2 (7·4-22·6) | 2·6 (1·2-5·6) | 257 | 54·4 (36·3-71·4) | 10·7 (4·8-22·2) | 56 |
|  |  | **Married 1+ children** | 50·2 (40·9-59·6) | 43·6 (35·0-52·7) | 209 | 71·4 (61·1-79·9) | 62·0 (51·5-71·6) | 138 |
| Indonesia (2012) | DHS | **Not married** | --- | --- | 17 | --- | --- | 16 |
|  |  | **Married no children** | 17·0 (12·5-22·9) | 16·7 (12·1-22·5) | 458 | 74·5 (62·7-83·6) | 73·1 (61·3-82·3) | 91 |
|  |  | **Married 1+ children** | 77·9 (72·4-82·6) | 77·3 (71·8-82·1) | 511 | 91·2 (87·9-93·7) | 90·5 (87·1-93·1) | 421 |
| Lao (2011) | MICS | **Not married** | 3·7 (0·9-13·9) | 3·7 (0·9-13·9) | 47 | 3·9 (1·0-14·7) | 3·9 (1·0-14·7) | 44 |
|  |  | **Married no children** | 18·0 (14·4-22·3) | 15·8 (12·4-20·1) | 523 | 48·0 (39·9-56·2) | 43·2 (35·0-51·9) | 178 |
|  |  | **Married 1+ children** | 33·2 (29·0-37·8) | 27·9 (23·8-32·3) | 644 | 65·1 (58·8-70·9) | 58·9 (52·1-65·3) | 294 |
| Mongolia (2013) | MICS | **Not married** | 42·6 (26·6-60·3) | 34·7 (20·7-51·9) | 37 | 45·7 (286-63·9) | 37·2 (22·2-55·1) | 35 |
|  |  | **Married no children** | 15·3 (6·2-33·1) | 15·3 (6·2-33·1) | 35 | --- | --- | 15 |
|  |  | **Married 1+ children** | 39·5 (25·7-55·3) | 37·0 (23·5-52·8) | 46 | 50·6 (33·6-67·4) | 47·3 (30·7-64·5) | 36 |
| Myanmar (2015) | DHS | **Not married** | --- | --- | 0 | --- | --- | 0 |
|  |  | **Married no children** | 48·5 (38·9-58·2) | 47·2 (37·6-56·9) | 145 | 73·5 (62·4-82·3) | 71·5 (60·1-80·7) | 89 |
|  |  | **Married 1+ children** | 63·6 (51·0-74·5) | 63·6 (51·0-74·5) | 90 | 74·9 (61·3-85·0) | 74·9 (61·3-85·0) | 75 |
| Philippines (2013) | DHS | **Not married** | 42·1 (26·6-59·4) | 15·6 (6·6-32·6) | 35 | 46·2 (29·1-64·2) | 17·1 (7·1-35·8) | 32 |
|  |  | **Married no children** | 8·4 (4·4-15·5) | 1·6 (0·4-6·3) | 124 | 23·8 (13·0-39·6) | 4·5 (1·1-16·9) | 43 |
|  |  | **Married 1+ children** | 53·4 (46·0-60·6) | 29·9 (24·0-36·6) | 195 | 64·1 (55·9-71·6) | 36·0 (29·1-43·5) | 158 |
| Timor Leste (2016) | DHS | **Not married** | --- | --- | 9 | --- | --- | 5 |
|  |  | **Married no children** | 2·2 (0·3-13·5) | 0 | 88 | 7·6 (1·0-40·3) | 0 | 21 |
|  |  | **Married 1+ children** | 15·9 (9·7-24·9) | 12·1 (7·0-20·0) | 140 | 37·5 (23·5-53·9) | 28·5 (16·5-44·6) | 58 |
| Vietnam (2010) | MICS | **Not married** | --- | --- | 4 | --- | --- | 4 |
|  |  | **Married no children** | 4·4 (1·4-13·0) | 2·2 (0·4-10·7) | 78 | --- | --- | 10 |
|  |  | **Married 1+ children** | 37·1 (26·2-49·5) | 28·1 (17·9-41·2) | 79 | 68·4 (55·4-78·9) | 53·7 (36·6-69·9) | 51 |
| **South Asia** | | | | | | | | |
| Bhutan (2010) | MICS | **Not married** | --- | --- | 9 | --- | --- | 7 |
|  |  | **Married no children** | 5·8 (3·3-10·1) | 5·8 (3·3-10·1) | 160 | 15·9 (8·9-26·7) | 15·9 (8·9-26·7) | 57 |
|  |  | **Married 1+ children** | 54·5 (44·6-64·1) | 54·5 (44·6-64·1) | 171 | 69·5 (58·8-78·5) | 69·5 (58·8-78·5) | 134 |
| India (2015) | DHS | **Not married** | 11·8 (5·6-22·9) | 10·3 (4·7-21·1) | 64 | 33·9 (15·8-58·6) | 29·7 (13·1-54·0) | 22 |
|  |  | **Married no children** | 9·6 (8·7-10·6) | 5·6 (4·9-6·3) | 11347 | 34·0 (31·4-36·7) | 19·7 (17·5-22·1) | 3183 |
|  |  | **Married 1+ children** | 25·8 (24·0-27·7) | 18·6 (17·0-20·2) | 5517 | 46·7 (44·0-49·4) | 33·5 (31·1-36·1) | 3000 |
| Nepal (2016) | DHS | **Not married** | --- | --- | 1 | --- | --- | 1 |
|  |  | **Married no children** | 15·0 (11·0-20·1) | 8·1 (4·9-13·2) | 407 | 34·1 (26·1-43·2) | 18·5 (11·5-28·3) | 201 |
|  |  | **Married 1+ children** | 32·7 (26·9-39·0) | 22·0 (17·3-27·9) | 334 | 43·8 (36·9-50·9) | 29·4 (22·9-36·9) | 253 |

--- not enough sample size; n<20
