# Supplementary material for: Contraception in adolescence: the influence of parity and marital status on contraceptive use in 73 low-and middle-income countries
Source: Reprod Health. 2019 Feb 21;16:21. doi: 10.1186/s12978-019-0686-9 (PMC6383262; doi:10.1186/s12978-019-0686-9)
Supplement: Supplementary file 5 — Contraceptive use prevalence and demand for family planning satisfied coverage with any and modern methods among female adolescents in Latin America & Caribbean countries. (DOCX 24 kb) [file 12978_2019_686_MOESM5_ESM.docx]

**Additional file 5. Contraceptive use prevalence and demand for family planning satisfied coverage with any and modern methods in Latin America & Caribbean countries.**

| **Country** | **Source** | **Status** | **CPR**  **% (95%CI)** | **mCPR**  **% (95%CI)** | **N** | **DFPS**  **% (95%CI)** | **mDFPS**  **% (95%CI)** | **N** |
| --- | --- | --- | --- | --- | --- | --- | --- | --- |
| Belize (2011) | MICS | **Not married** | 57·7 (42·3-71·7) | 57·7 (42·3-71·7) | 47 | 63·3 (48·0-76·3) | 63·3 (48·0-76·3) | 44 |
|  |  | **Married no children** | 18·1 (10·0-30·6) | 15·7 (8·2-27·8) | 59 | 38·3 (22·1-57·7) | 33·1 (17·9-53·1) | 28 |
|  |  | **Married 1+ children** | 51·6 (39·5-63·5) | 50·3 (38·7-61·9) | 73 | 62·5 (48·2-74·9) | 60·9 (47·1-73·1) | 60 |
| Bolivia (2008) | DHS | **Not married** | 67·3 (57·4-75·9) | 43·6 (33·6-54·0) | 131 | 69·8 (59·7-78·2) | 45·1 (34·9-55·8) | 128 |
|  |  | **Married no children** | 24·7 (17·1-34·3) | 10·6 (6·3-17·3) | 167 | 38·3 (27·6-50·4) | 16·5 (9·7-26·6) | 107 |
|  |  | **Married 1+ children** | 48·9 (42·9-54·9) | 32·3 (27·0-38·2) | 345 | 57·1 (50·4-63·6) | 37·8 (31·7-44·3) | 290 |
| Colombia (2015) | DHS | **Not married** | 81·6 (78·0-84·7) | 76·7 (72·8-80·1) | 924 | 83·2 (79·7-86·2) | 78·2 (74·4-81·6) | 906 |
|  |  | **Married no children** | 39·7 (32·9-46·9) | 36·4 (29·8-43·5) | 389 | 58·2 (50·3-65·6) | 53·4 (45·6-61·0) | 272 |
|  |  | **Married 1+ children** | 80·5 (76·3-84·1) | 75·5 (71·1-79·5) | 657 | 85·8 (81·9-89·0) | 80·6 (76·3-84·2) | 614 |
| Costa Rica (2011) | MICS | **Not married** | 54·6 (39·2-69·1) | 54·6 (39·2-69·1) | 89 | 59·3 (43·4-73·4) | 59·3 (43·4-73·4) | 82 |
|  |  | **Married no children** | 51·2 (30·4-71·6) | 51·2 (30·4-71·6) | 60 | 69·7 (45·9-86·2) | 69·7 (45·9-86·2) | 44 |
|  |  | **Married 1+ children** | 75·0 (57·1-87·1) | 75·0 (57·1-87·1) | 80 | 81·1 (62·8-91·6) | 81·1 (62·8-91·6) | 76 |
| Cuba (2014) | MICS | **Not married** | 94·0 (88·5-96·9) | 94·0 (88·5-96·9) | 231 | 94·2 (88·7-97·1) | 94·2 (88·7-97·1) | 227 |
|  |  | **Married no children** | 51·0 (31·3-70·4) | 51·0 (31·3-70·4) | 52 | 65·4 (40·1-84·2) | 65·4 (40·1-84·2) | 41 |
|  |  | **Married 1+ children** | 73·3 (51·7-87·6) | 73·3 (51·7-87·6) | 144 | 82·8 (58·0-94·4) | 82·8 (58·0-94·4) | 131 |
| Dominican Republic (2014) | MICS | **Not married** | 58·8 (51·7-65·5) | 58·8 (51·7-65·5) | 549 | 64·5 (57·2-71·4) | 64·5 (57·2-71·4) | 501 |
|  |  | **Married no children** | 35·1 (28·1-42·9) | 33·9 (27·0-41·6) | 309 | 61·8 (52·2-70·6) | 59·8 (50·1-68·9) | 179 |
|  |  | **Married 1+ children** | 65·9 (61·3-70·2) | 64·7 (60·0-69·1) | 968 | 72·8 (68·3-76·9) | 71·5 (66·9-75·7) | 878 |
| El Salvador (2014) | MICS | **Not married** | 48·2 (36·0-60·7) | 43·9 (32·0-56·5) | 112 | 53·9 (40·5-66·7) | 49·0 (36·0-62·2) | 101 |
|  |  | **Married no children** | 33·8 (24·3-44·8) | 32·3 (22·8-43·4) | 113 | 52·4 (38·4-66·0) | 50·4 (36·5-64·2) | 66 |
|  |  | **Married 1+ children** | 76·8 (70·8-81·8) | 73·1 (67·0-78·5) | 386 | 83·1 (77·8-87·3) | 79·9 (74·2-84·6) | 358 |
| Guatemala (2014) | DHS | **Not married** | 55·8 (45·2-66·0) | 41·7 (31·7-52·4) | 128 | 64·0 (52·8-73·9) | 47·8 (37·0-58·8) | 113 |
|  |  | **Married no children** | 15·5 (11·8-19·9) | 9·1 (6·3-13·0) | 427 | 43·7 (34·5-53·4) | 25·7 (18·2-35·0) | 162 |
|  |  | **Married 1+ children** | 54·1 (49·6-58·5) | 43·6 (39·1-48·2) | 732 | 70·1 (65·3-74·6) | 56·5 (51·4-61·4) | 564 |
| Guyana (2014) | MICS | **Not married** | 21·9 (7·3-50·0) | 21·9 (7·3-50·0) | 28 | 22·7 (7·7-50·9) | 22·7 (7·7-50·9) | 27 |
|  |  | **Married no children** | 4·6 (2·1-9·6) | 3·8 (1·6-8·5) | 139 | 6·5 (2·9-13·6) | 5·3 (2·2-12·0) | 102 |
|  |  | **Married 1+ children** | 25·1 (17·4-34·6) | 25·1 (17·4-34·6) | 143 | 31·8 (22·4-42·9) | 31·8 (22·4-42·9) | 116 |
| Haiti (2012) | DHS | **Not married** | 30·8 (23·8-38·8) | 28·2 (21·3-36·2) | 285 | 32·5 (25·3-40·7) | 29·7 (22·6-37·9) | 271 |
|  |  | **Married no children** | 12·4 (7·4-20·1) | 9·9 (5·6-17·1) | 156 | 16·2 (9·9-25·5) | 13·0 (7·4-21·8) | 122 |
|  |  | **Married 1+ children** | 33·7 (26·0-42·2) | 32·1 (24·5-40·7) | 265 | 39·2 (30·6-48·6) | 37·4 (28·8-46·8) | 224 |
| Honduras (2011) | DHS | **Not married** | 75·5 (67·1-82·4) | 56·1 (47·5-64·3) | 171 | 80·7 (73·0-86·5) | 59·9 (51·4-67·9) | 162 |
|  |  | **Married no children** | 34·0 (28·6-39·8) | 27·4 (22·6-32·9) | 482 | 65·8 (57·7-73·0) | 53·1 (45·2-60·9) | 236 |
|  |  | **Married 1+ children** | 70·2 (66·3-73·9) | 64·2 (60·0-68·1) | 755 | 79·9 (76·0-83·3) | 73·0 (68·7-76·8) | 653 |
| Mexico (2015) | MICS | **Not married** | 22·7 (13·2-36·1) | 22·7 (13·2-36·1) | 93 | 23·9 (13·6-38·4) | 23·9 (13·6-38·4) | 84 |
|  |  | **Married no children** | 28·6 (16·2-45·2) | 28·6 (16·2-45·2) | 71 | 51·0 (29·3-72·3) | 51·0 (29·3-72·3) | 35 |
|  |  | **Married 1+ children** | 61·9 (54·0-69·3) | 60·8 (52·8-68·3) | 337 | 67·6 (58·7-75·4) | 66·6 (57·6-74·5) | 302 |
| Panama (2013) | MICS | **Not married** | 54·4 (40·3-67·9) | 52·2 (38·2-65·8) | 141 | 55·0 (40·7-68·6) | 52·7 (38·6-66·4) | 137 |
|  |  | **Married no children** | 24·4 (15·3-36·7) | 18·3 (10·6-29·6) | 130 | 36·9 (23·3-53·0) | 27·6 (16·1-429) | 94 |
|  |  | **Married 1+ children** | 41·0 (32·3-50·4) | 34·0 (25·6-43·7) | 283 | 48·4 (36·5-60·4) | 40·3 (29·2-52·5) | 238 |
| Peru (2012) | DHS | **Not married** | 87·7 (81·8-91·9) | 63·1 (56·6-69·2) | 269 | 89·1 (83·6-93·0) | 62·4 (54·6-69·6) | 266 |
|  |  | **Married no children** | 46·0 (37·8-54·5) | 29·4 (22·2-37·7) | 218 | 63·0 (53·4-71·8) | 40·2 (31·1-50·1) | 161 |
|  |  | **Married 1+ children** | 78·7 (73·2-83·3) | 61·4 (53·6-68·7) | 386 | 88·5 (83·8-92·0) | 67·6 (61·0-73·6) | 359 |
| Suriname (2010) | MICS | **Not married** | 45·1 (36·3-54·3) | 44·9 (36·1-54·1) | 171 | 54·1 (44·2-63·6) | 53·9 (44·0-63·6) | 76 |
|  |  | **Married no children** | 45·5 (30·0-62·6) | 45·5 (30·0-62·6) | 48 | 59·8 (39·9-76·9) | 59·8 (39·9-76·9) | 36 |
|  |  | **Married 1+ children** | 40·0 (29·1-52·1) | 40·0 (29·1-52·1) | 98 | 49·3 (36·4-62·3) | 49·3 (36·4-62·3) | 140 |
| Trinidad and Tobago (2006) | MICS | **Not married** | 45·4 (33·1-58·4) | 45·4 (33·1-58·4) | 61 | 55·6 (41·3-69·1) | 55·6 (41·3-69·1) | 50 |
|  |  | **Married no children** | 36·2 (20·3-55·9) | 32·3 (17·5-51·7) | 28 | --- | --- | 19 |
|  |  | **Married 1+ children** | 48·1 (27·8-69·1) | 48·1 (27·8-69·1) | 21 | 50·3 (29·0-71·5) | 50·3 (29·0-71·5) | 20 |

--- not enough sample size; n<20
